# Case Report: Severe mixed autoimmune hemolytic anemia associated with acute cytomegalovirus infection in an immunocompetent adult

**DOI:** 10.3389/fimmu.2026.1913000

**Published:** 2026-09-15

**Authors:** Junjie Hou, Yanhong Ding, Huiyao Chen, Min Liao, Chun Feng, Jihao Zhou, Guoqiang Li, Peng Ke, Feichang Du

**Affiliations:** 1 Department of Hematology, Shenzhen People’s Hospital (The Second Clinical Medical College, Jinan University; The First Affiliated Hospital, Southern University of Science and Technology), Shenzhen, Guangdong, China; 2 Department of Laboratory Medicine, Shenzhen People’s Hospital (The Second Clinical Medical College, Jinan University; The First Affiliated Hospital, Southern University of Science and Technology), Shenzhen, Guangdong, China; 3 Shenzhen Bao’an District Central Blood Station, Shenzhen, China

**Keywords:** cytomegalovirus, extended direct antiglobulin test, immunocompetent host, mixed autoimmune hemolytic anemia, thermal amplitude

## Abstract

**Background:**

Cytomegalovirus (CMV)-associated hemolytic anemia (HA) is rare in immunocompetent hosts and may present with heterogeneous serologic phenotypes, including direct antiglobulin test (DAT)-negative hemolysis, warm autoimmune hemolytic anemia (AIHA), cold antibody-mediated AIHA, and rarely mixed AIHA. Because mixed AIHA requires evidence of both warm autoantibodies and clinically significant cold autoantibody activity, this phenotype may be underrecognized without comprehensive immunohematologic testing.

**Case presentation:**

We report a case of CMV-associated HA that was diagnosed as mixed AIHA by comprehensive immunohematologic assessment and successfully managed with combination therapy. A 59-year-old immunocompetent man presented with a 7-day history of fever, sore throat, and fatigue. Initial evaluation revealed marked hepatocellular and cholestatic liver injury, positive CMV IgM with negative CMV IgG, and serum CMV DNA of 2.28 × 10^5^ IU/mL. After exclusion of common viral and autoimmune causes, acute CMV hepatitis was clinically diagnosed. On hospital day 4, he developed dark brown urine, with hemoglobin falling to 40 g/L and laboratory evidence of acute hemolysis. Extended DAT revealed IgG, IgA, IgM, and C3d coating of erythrocytes, and cold agglutinin testing identified an IgM cold agglutinin with a titer of 1:128 and thermal amplitude extending to 37 °C, supporting severe CMV-associated mixed AIHA. After antiviral therapy, immunomodulatory and supportive treatment, hemolysis was controlled, and hemoglobin stabilized without further transfusion.

**Conclusions:**

CMV-associated HA may rarely present as severe mixed AIHA in immunocompetent adults. In severe or atypical cases, comprehensive immunohematologic evaluation beyond routine DAT, including extended DAT, cold agglutinin titration, and thermal amplitude testing, is essential for accurate subtyping. In severe cases, early integrated therapy targeting both CMV replication and immune-mediated hemolysis may be required.

## Introduction

Autoimmune hemolytic anemia (AIHA) is an acquired immune cytopenia caused by autoantibody-mediated red cell destruction, which can be classified into primary and secondary according to the etiology. In addition, according to the thermal characteristics and immunohematologic features of the causative autoantibodies, AIHA can also be subdivided into warm AIHA (wAIHA), cold agglutinin disease (CAD), mixed AIHA, paroxysmal cold hemoglobinuria, and atypical AIHA (1). Among these, wAIHA is the predominant subtype, accounting for more than two-thirds of adult cases, whereas mixed AIHA accounts for fewer than 10% of AIHA cases but remains clinically significant (2). Mixed AIHA is characterized by the coexistence of warm-reactive IgG and pathogenic cold-reactive IgM autoantibodies, leading to both Fc receptor-mediated extravascular hemolysis and complement-mediated red cell injury (3, 4). This dual serologic profile creates a diagnostic challenge, because IgG and C3d positivity on the direct antiglobulin test (DAT) alone does not establish mixed AIHA, as complement deposition may also occur in wAIHA (4). Therefore, the diagnosis requires evidence of both a warm autoantibody and a clinically significant cold agglutinin, supported by monospecific DAT, serum or eluate studies, cold agglutinin titer, and thermal amplitude testing (4–6). Accurate classification is clinically relevant because mixed AIHA is often severe, prone to misclassification, and frequently requires transfusion support and combination therapy (7, 8).

Cytomegalovirus (CMV), a ubiquitous β-herpesvirus within the Herpesviridae family, infects a large proportion of the general population and produces a clinical spectrum that is largely shaped by host immune status. In immunocompetent individuals, primary CMV infection is usually asymptomatic or manifests as a self-limited mononucleosis-like syndrome. By contrast, in immunocompromised patients, including transplant recipients, individuals with advanced human immunodeficiency virus (HIV) infection, and patients receiving intensive immunosuppression, CMV may cause invasive end-organ disease through direct viral cytopathic injury and immune dysregulation, involving the gastrointestinal tract, retina, central nervous system, respiratory tract, liver, and hematopoietic system (9–11).

Severe CMV-associated hemolytic anemia (HA) is uncommon, particularly in immunocompetent adults. Previous reports on CMV-associated HA include both DAT-positive AIHA and DAT-negative hemolysis. Among the former, most reported cases have shown a warm autoantibody pattern, whereas CMV-associated mixed AIHA has been only sporadically described (11, 12). Here, we report a severe case of mixed AIHA associated with acute CMV hepatitis in an apparently immunocompetent adult, with targeted immunohematologic evaluation supporting both warm and cold autoantibody components.

## Case presentation

A 59-year-old man was admitted to the hospital with a 7-day history of fever, sore throat, and fatigue. He had no significant past medical history and was not receiving immunosuppressive therapy. Complete blood count (CBC) tests revealed white blood cell count (WBC) 12.42 × 10^9^/L (normal, 3.5-9.5 × 10^9^/L), hemoglobin (Hb) 127 g/L (normal, 130–170 g/L) and platelet count (PLT) 294 × 10^9^/L (normal, 125-350 × 10^9^/L). Peripheral blood (PB) smear showed monocytosis and a few reactive lymphocytes. CMV serology showed positive IgM with negative IgG, suggestive of acute infection. Polymerase chain reaction (PCR) testing detected a serum CMV DNA viral load of 2.28 × 10^5^ IU/mL. Abdominal ultrasonography showed hepatomegaly (right hepatic lobe oblique diameter, 162 mm) with diffusely decreased and heterogeneous hepatic echogenicity, consistent with acute hepatic parenchymal involvement. Mild gallbladder wall and biliary duct wall thickening were also observed, with a small amount of lower abdominal ascites, while the pancreas, spleen, and portal venous system were unremarkable. Other laboratory test results are shown in **Table 1**. Based on the examination results above, the patient was clinically diagnosed with acute CMV hepatitis.

**Table 1:** Investigations of the patient.

Investigation	Result
Clinical biochemistry	Clinical biochemistry results revealed total bilirubin (TBIL) 485.8 μmol/L (normal, 1.7-20.0 μmol/L), direct bilirubin (DBIL) 284.1 μmol/L (normal, 0.0-6.0 μmol/L), indirect bilirubin (IBIL) 201.7 μmol/L (normal, 1.7-16.0 μmol/L), alanine aminotransferase (ALT) 1435 U/L (normal, 13-45 U/L), aspartate aminotransferase (AST) 2095 U/L (normal, 7-40 U/L), γ-glutamyl transferase (GGT) 561 U/L (normal, 10-60 U/L), alkaline phosphatase (ALP) 394 U/L (normal, 45-125 U/L), lactate dehydrogenase (LDH) 2982 U/L (normal, <248 U/L), creatinine (Cr) 85 μmol/L (normal, 59-104 μmol/L), and ceruloplasmin 0.534 g/L (normal, 0.2-0.6 g/L).
Coagulation profile	Coagulation studies revealed a prolonged prothrombin time (PT) of 22.4 s (normal, 11.0-14.5 s), an elevated international normalized ratio (INR) of 1.96 (normal, 0.8-1.2), decreased prothrombin activity of 38% (normal, 70-130%), a prolonged activated partial thromboplastin time (APTT) of 64.5 s (normal, 28-43 s), a mildly elevated fibrinogen level of 4.20 g/L (normal, 2.0-4.0 g/L), and an elevated D-dimer level of 1.50 μg/mL (normal, 0-0.5 μg/mL).
Viral serologies	Serologic testing for other viral infections was negative, including hepatitis A virus IgM, hepatitis E virus IgM, hepatitis B surface antigen, hepatitis B core IgM antibody, hepatitis C virus antibody, human immunodeficiency virus (HIV) p24 antigen, and HIV-1/2 antibodies. Epstein-Barr virus viral capsid antigen (EBV VCA) IgM was negative, whereas EBV nuclear antigen (EBNA) IgG was positive.
Autoimmune panel	Serologic evaluation for autoimmune liver disease and systemic rheumatic disorders showed no detectable antinuclear antibodies, extractable nuclear antigen antibodies, anticardiolipin antibodies, anti-smooth muscle antibodies, anti-liver-kidney microsomal antibodies, or antimitochondrial antibodies.

On hospital day 1, supportive management was initiated and included hepatoprotective therapy with magnesium isoglycyrrhizinate, treatment of hyperbilirubinemia with ademetionine 1,4-butanedisulfonate, and artificial liver support with a dual plasma molecular adsorption system (DPMAS). Intravenous (IV) ganciclovir was subsequently started on hospital day 3 at 5 mg/kg every 12 hours. On hospital day 4, the patient abruptly developed dark brown urine, accompanied by marked worsening of fatigue and other symptoms. A CBC revealed severe anemia, with Hb 40 g/L, mean corpuscular volume (MCV) 98.3 fL, mean corpuscular hemoglobin (MCH) 33.3 pg, mean corpuscular hemoglobin concentration (MCHC) 339 g/L, absolute reticulocyte count (ARC) 145 × 10^9^/L, PLT 273 × 10^9^/L, and WBC 14.58 × 10^9^/L. PB smear showed leukocytosis with prominent granulocytic left shift, numerous spherocytes, polychromasia, nucleated red blood cells, and occasional erythrophagocytosis by monocytes. Urine analysis showed dark brown urine with urinary bilirubin 3+ (normal, negative), protein 2+ (normal, negative), and urine occult blood 3+ (normal, negative), and ketones, urobilinogen, nitrite, and leukocyte esterase were negative. Other remarkable laboratory findings included total bilirubin (TBIL) 926 μmol/L, direct bilirubin (DBIL) 427.4 μmol/L, indirect bilirubin (IBIL) 498.6 μmol/L, alanine aminotransferase (ALT) 310 U/L, aspartate aminotransferase (AST) 371 U/L, lactate dehydrogenase (LDH) 3535 U/L, creatinine (Cr) 113 μmol/L, haptoglobin (HPT) <0.2 g/L (normal, 0.2-2.0), and a positive standard DAT (polyspecific, anti-IgG, and anti-C3d). Extended DAT identified IgG (4+), IgA (4+), IgM (3+), and C3d (3+) reactivity on the patient’s erythrocytes, whereas C3c and the negative control were negative (**Figure 1A**). Further immunohematologic evaluation showed a positive indirect antiglobulin test (IAT). The eluate prepared from the patient’s erythrocytes reacted broadly with reagent red blood cells. No definite clinically significant alloantibody was identified. In addition, an IgM cold agglutinin was detected at a titer of 1:128, with a thermal amplitude extending to 37 °C. Evaluation for other potential secondary causes revealed no evidence of a lymphoproliferative disorder on computed tomography or bone marrow examination. Autoimmune serologic testing was negative, and infectious disease testing provided no evidence of acute EBV infection, hepatitis A, B, C, or E virus infection, or HIV infection (**Table 1** and **Supplementary Table 1**). Taken together, these findings supported the diagnosis of severe mixed AIHA associated with acute CMV infection.

**Figure 1:**
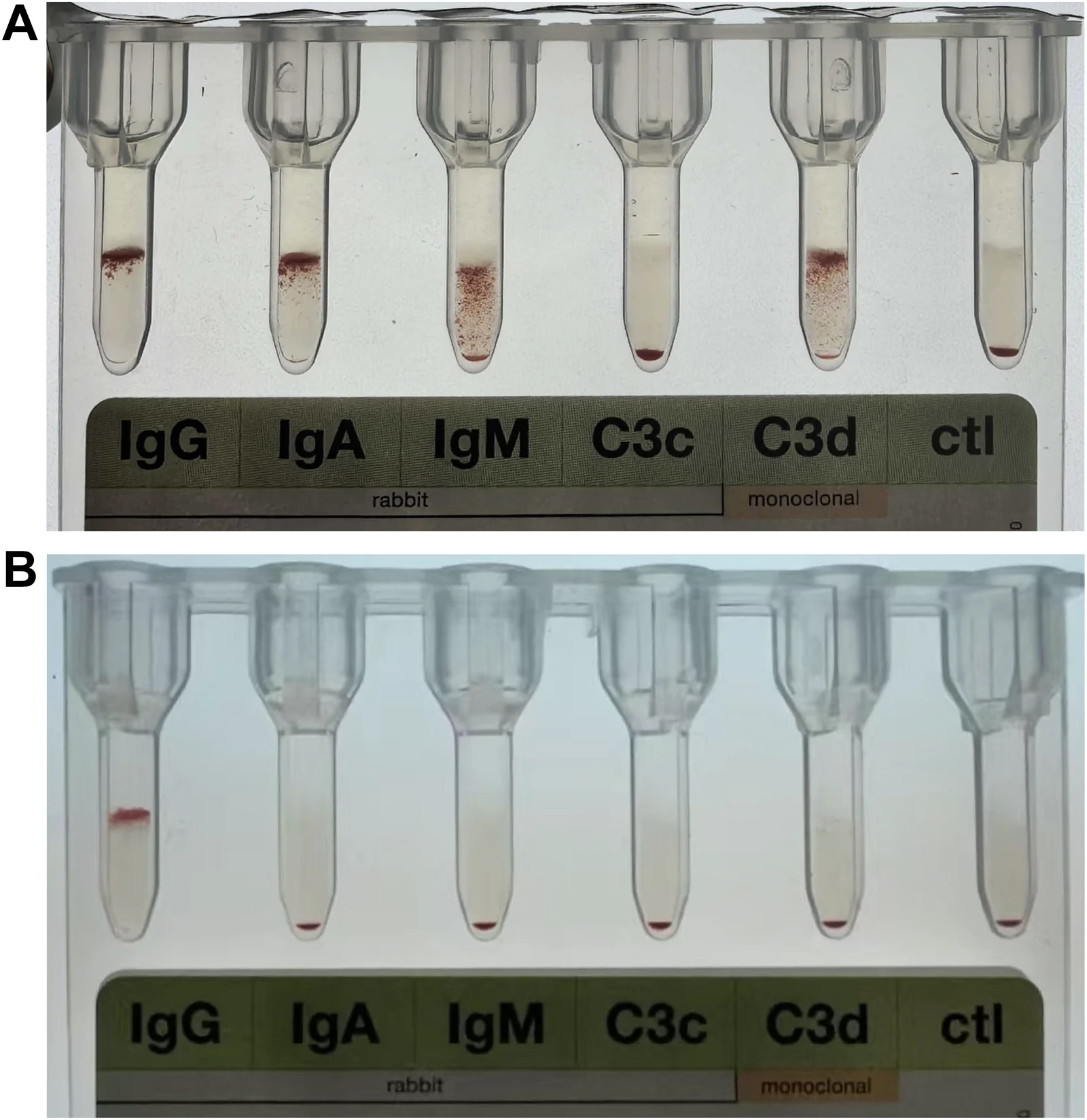
Serial changes in extended direct antiglobulin test (DAT) results during hospitalization. **(A)** At diagnosis, extended DAT demonstrated erythrocyte-bound IgG, IgA, IgM, and C3d, with negative C3c and control results. **(B)** Repeat extended DAT on hospital day 21 showed persistent isolated IgG positivity, with loss of IgA, IgM, and C3d reactivity.

On hospital day 5, when immune-mediated hemolysis was confirmed, dexamethasone therapy at 0.15 mg/kg/day was initiated immediately, and the patient underwent plasma exchange and was transfused with 4 units of leukocyte-reduced red blood cells (RBCs). On the same day, intravenous immunoglobulin (IVIG) was initiated at 0.4 g/kg/day and continued for 5 consecutive days. On hospital day 6, the patient’s Hb level increased to 69 g/L, but severe anemia persisted. A repeat plasma exchange was performed, and the patient was transfused with 4 units of leukocyte-reduced RBCs. Subsequently, plasma exchange was performed intermittently. On hospital day 9, the Hb level had peaked at 104 g/L after transfusion support, followed by a gradual decline to 74 g/L over the next 4 days. On hospital day 14, in the absence of further transfusion support, the Hb level gradually increased from 74 to 93 g/L. Over the subsequent week, in the absence of further RBC transfusion support, intermittent plasma exchange was performed for the treatment of immune-mediated hemolysis and hyperbilirubinemia, and the Hb level gradually increased to 107 g/L. On hospital day 21, repeat extended DAT demonstrated persistent strong IgG positivity (4+), whereas all other tested markers had converted to negative (**Figure 1B**). On the same day, intravenous dexamethasone was tapered by 50% to 0.075 mg/kg/day. Four days later, repeat laboratory testing revealed Hb of 113 g/L, TBIL of 100.3 μmol/L, DBIL of 84.3 μmol/L, IBIL of 16.0 μmol/L, LDH of 297 U/L, ALT of 164 U/L, and AST of 104 U/L. The serum CMV DNA viral load was 1.38 × 10^3^ IU/mL. On the same day, the patient was discharged from our hospital and transferred to a specialized infectious diseases hospital for continued management of CMV hepatitis. The patient’s clinical course, including interventions and significant laboratory value trends, is depicted in **Figure 2** and **Supplementary Table 2**.

**Figure 2:**
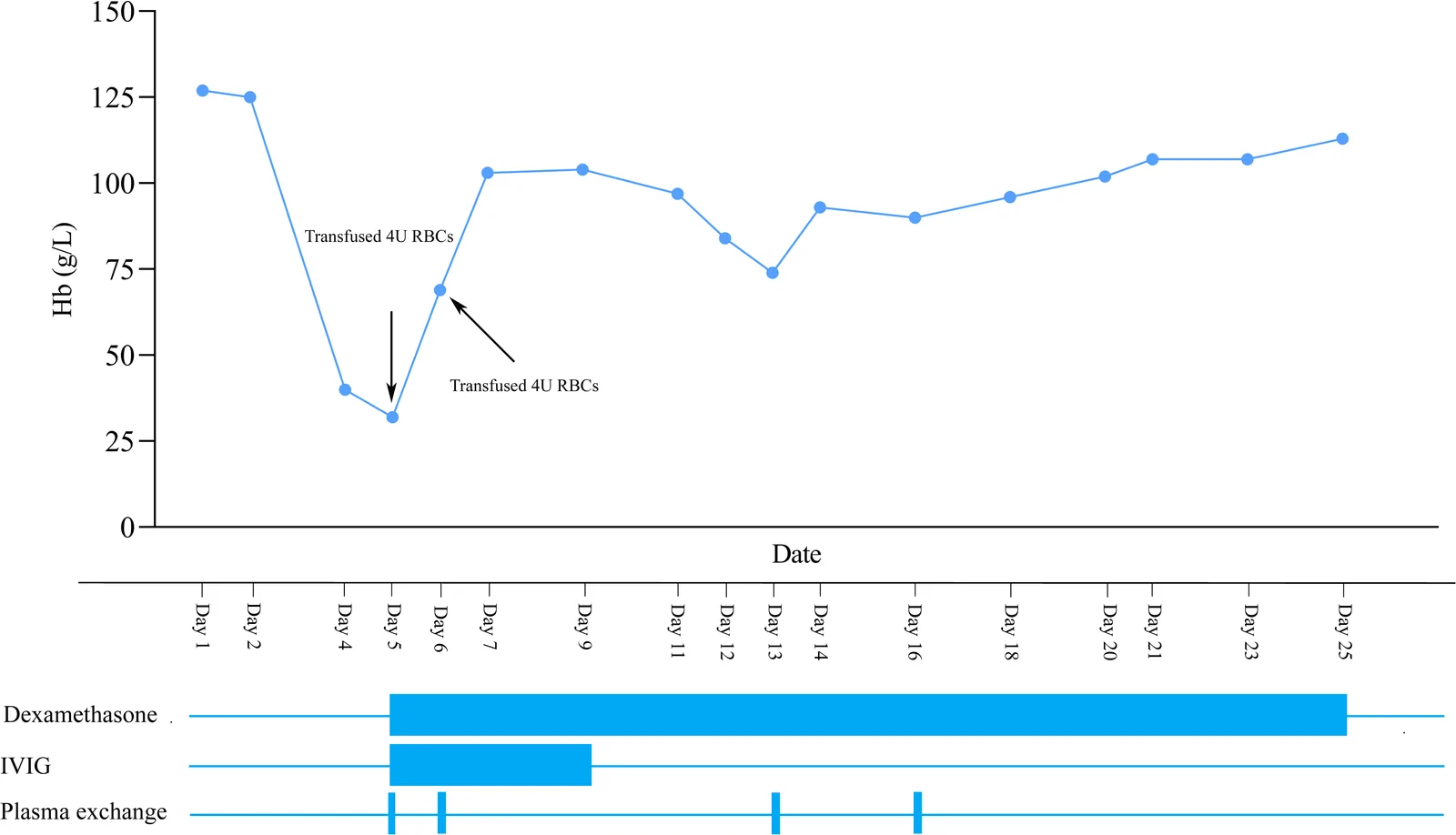
Hemoglobin trajectory and AIHA-directed interventions during hospitalization. Points indicate serial hemoglobin concentrations. The arrows indicate transfusion of 4 units of leukocyte-reduced RBCs on hospital days 5 and 6. Therapeutic plasma exchange was performed on hospital days 5, 6, 13, and 16. On hospital days 5 and 6, plasma exchange was completed before intravenous immunoglobulin (IVIG) administration. Dexamethasone was initiated on hospital day 5 and its dose was reduced on hospital day 21. IVIG was administered on hospital days 5-9.

## Discussion

CMV is a ubiquitous member of the family Herpesviridae, with adult seroprevalence rates generally ranging from 60% to 90% worldwide (13). Clinical manifestations of CMV infection differ substantially according to host immune status. In immunocompetent individuals, CMV infection is usually asymptomatic. In contrast, immunocompromised patients may develop clinically apparent CMV disease with multiorgan involvement, including the respiratory, gastrointestinal, and hematologic systems (9–11). Among 290 reported cases of severe CMV infection in apparently immunocompetent adults, gastrointestinal and neurologic manifestations predominated, while hematologic manifestations were uncommon (8.6%, 25/290). HA was particularly rare, occurring in only 1.7% of cases (14). Beyond these early aggregate data, subsequent reports of CMV-associated HA in immunocompetent individuals have remained largely limited to isolated cases and small case-based reviews (11, 15, 16). Therefore, CMV-associated HA is a rare manifestation of CMV infection in immunocompetent hosts.

The clinical expression of CMV-associated HA is also highly heterogeneous. At the milder end of the clinical spectrum, hemolysis may remain subclinical, with abnormalities detectable only on laboratory testing and no overt clinical manifestations. In the study by Horwitz et al., laboratory evidence of hemolysis was found in 9 of 20 patients with CMV mononucleosis, none of whom had overt hemolysis-related symptoms (17). This observation suggests that mild CMV-associated hemolysis may be underrecognized, particularly when hemolytic testing is not routinely performed. At the other end of the spectrum, CMV-associated HA may present as acute, severe, and potentially life-threatening, including cases of AIHA (15, 16). Our patient exemplifies the severe immune-mediated end of this spectrum, with an abrupt and marked Hb decline, pronounced biochemical hemolysis, and serologic evidence supporting mixed AIHA, accompanied by clinically diagnosed acute CMV hepatitis. This dual presentation underscores that CMV-associated HA may range from subclinical laboratory abnormalities to fulminant hemolytic crises with concurrent CMV-associated organ involvement. These observations suggest that CMV-associated HA should not be considered only after overt hemolytic crises develop. In patients with acute CMV infection, even a subtle decline in Hb or early laboratory evidence of hemolysis may warrant serial monitoring, thereby reducing the likelihood of delayed recognition of clinically significant anemia.

Beyond this variability in clinical severity, CMV-associated HA also shows substantial diversity in laboratory and immunohematologic profiles. Previously reported cases of CMV-associated hemolytic anemia demonstrate a heterogeneous serologic spectrum, including DAT-negative hemolysis, DAT-positive wAIHA, and cold antibody-mediated hemolysis, whereas mixed AIHA has been described only rarely (12, 15–17). This diversity likely reflects genuine biological differences in the mechanisms of hemolysis. CMV-associated hemolysis has been reported even when routine autoimmune testing was negative, suggesting that DAT-undetectable immune injury or nonimmune red cell injury may occur in some patients (15). However, this diversity may also be influenced by incomplete immunohematologic testing. In standard DAT, false-negative results may occur when the density of erythrocyte-bound IgG is below the assay detection threshold or when hemolysis is mediated by IgA or IgM antibodies that are not directly detected by conventional anti-IgG and anti-C3 reagents (18). In addition, complement deposition may also be observed in wAIHA, and the clinical significance of cold agglutinins depends on both their titer and thermal amplitude (4, 6). Therefore, reliance on a positive standard DAT alone, without subsequent extended DAT, serum and/or eluate studies, cold agglutinin titration, and thermal amplitude assessment, may lead to under-recognition or misclassification of cold antibody-mediated hemolysis or mixed AIHA. In our patient, routine DAT established immune-mediated hemolysis, whereas extended DAT, eluate reactivity, and cold agglutinin thermal amplitude testing supported the coexistence of warm and clinically significant cold autoantibody components. These findings underscore the importance of comprehensive immunohematologic evaluation in severe or atypical CMV-associated HA.

Beyond immunohematologic classification, assessment of the likely trigger requires careful consideration of alternative causes of hemolysis. Drug-induced immune hemolytic anemia was considered because severe hemolysis developed approximately one day after the initiation of ganciclovir therapy. However, ganciclovir, magnesium isoglycyrrhizinate, and ademetionine 1,4-butanedisulfonate were continued despite subsequent control of hemolysis, making a classic drug-dependent mechanism less likely. Because drug-dependent antibody testing was not performed, a potential drug-related contribution could not be completely excluded (16, 19). Normal G6PD activity, together with the strongly positive DAT and demonstrable warm- and cold-reactive autoantibodies, made isolated oxidative hemolysis due to G6PD deficiency less likely. In addition, the temporal relationship to virologically documented acute CMV infection and the absence of evidence for lymphoproliferative disease, autoimmune disease, or the alternative infections tested also support a plausible association between CMV infection and mixed AIHA in this patient. However, this inference is based on temporal association and exclusion of other identified causes rather than direct evidence of causality.

AIHA management should be individualized according to disease severity, hemoglobin kinetics, immunohematologic subtype, and secondary triggers. In severe AIHA, clinically indicated RBC transfusion should not be withheld or delayed. Disease-directed therapy may include corticosteroids, rituximab, other immunosuppressive agents, IVIG, and plasma exchange in selected cases (4, 5, 7). However, no standardized treatment algorithm exists for CMV-associated AIHA due to its marked heterogeneity (9, 11, 20). For patients with only laboratory evidence of hemolysis and no overt hemolysis-related symptoms, observation with serial monitoring may be sufficient (17). When hemolysis is the predominant manifestation in the absence of definite CMV disease, some patients recover spontaneously with observation alone, whereas others recover with RBC transfusion support alone, without additional immunomodulatory or antiviral therapy (15, 21). By contrast, when severe CMV-associated AIHA is accompanied by extensive CMV disease or additional immune cytopenias, prompt combination therapy directed at both CMV infection and hemolysis is often required (22). Nevertheless, targeted antiviral therapy with ganciclovir or valganciclovir has been considered appropriate for severe CMV disease in immunocompetent adults, despite the limited evidence base (23). Although direct comparative data among CMV-associated AIHA subtypes are lacking, mixed AIHA as a serologic subtype has been associated with severe onset, treatment refractoriness, transfusion support, and the need for multiple therapeutic modalities. This may be particularly relevant when mixed AIHA occurs in the context of active CMV disease or immunocompromised states (6, 8, 12, 24, 25). The 2023 American Society for Apheresis (ASFA) guidelines classify therapeutic plasma exchange as category II, grade 2C for severe cold agglutinin disease and category III, grade 2C for severe warm AIHA, while mixed AIHA is not separately categorized (26). Given the fulminant, transfusion-requiring hemolysis and the coexistence of warm and clinically significant cold-reactive autoantibodies, therapeutic plasma exchange was selected as individualized rescue and bridging therapy as immunomodulatory treatment took effect (26, 27). During multimodal treatment with ganciclovir, dexamethasone, IVIG, plasma exchange, and transfusion support, hemolysis was controlled. However, because these interventions were administered in close temporal proximity, the contribution of any individual treatment cannot be determined. This clinical course suggests that severe CMV-associated mixed AIHA may warrant individualized multimodal management.

Notably, in our patient, despite clinical control of hemolysis, repeat extended DAT remained strongly positive for IgG, whereas IgA, IgM, and C3d had become negative. This serologic pattern may have two explanations. First, given the strong IgG DAT positivity and panreactive eluate before IVIG administration, persistent IgG reactivity may reflect residual warm autoantibody-mediated erythrocyte sensitization, while the disappearance of IgM and C3d is consistent with attenuation of the cold antibody/complement-mediated component (28). Second, recent high-dose IVIG may also have contributed to persistent IgG reactivity through passive red-cell coating by IVIG-derived IgG isoagglutinins, particularly in this group AB patient (29, 30). However, no post-IVIG eluate or subsequent DAT results were available. Therefore, these mechanisms could not be further distinguished. Accordingly, post-treatment DAT findings, particularly after IVIG, should be interpreted alongside hemoglobin kinetics and biochemical markers of hemolysis rather than as an isolated indicator of ongoing hemolysis (31).

Several limitations should be acknowledged. First, despite the close temporal association and the absence of another identified secondary trigger, a definitive causal relationship between CMV infection and mixed AIHA cannot be established from a single case. Second, acute CMV hepatitis was clinically diagnosed on the basis of acute CMV serology, a high serum CMV DNA viral load, exclusion of common viral and autoimmune causes, and compatible imaging findings, but liver histopathology was not available. Third, as a single case in a rare and heterogeneous clinical setting, the generalizability of the observed clinical course and serologic evolution is limited. Fourth, limited follow-up after transfer to a specialized infectious diseases hospital precluded subsequent DAT and post-IVIG eluate testing and limited assessment of the durability of remission.

## Conclusions

We report a case of severe mixed AIHA associated with acute CMV hepatitis in an apparently immunocompetent adult. Comprehensive immunohematologic assessment, including extended DAT, IAT, eluate testing, cold agglutinin titration, and thermal amplitude testing, supported the diagnosis of severe CMV-associated mixed AIHA. Hemolysis was controlled after anti-CMV therapy, RBC transfusion, glucocorticoids, IVIG, and plasma exchange. This case suggests that unexplained anemia or early laboratory evidence of hemolysis in patients with acute CMV infection should prompt consideration of CMV-associated AIHA, even without overt immunosuppression. Furthermore, in suspected mixed AIHA, standard DAT alone is insufficient for subtype classification, and targeted additional testing should be considered. Although no standardized treatment strategy has been established for CMV-associated hemolytic anemia, severe CMV-associated mixed AIHA may require integrated therapy targeting both viral replication and immune-mediated hemolysis.

## Data Availability

The raw data supporting the conclusions of this article will be made available by the authors, without undue reservation.
