# Supplementary material for: Case Report: Severe mixed autoimmune hemolytic anemia associated with acute cytomegalovirus infection in an immunocompetent adult
Source: Front Immunol. 2026 Sep 15;17:1913000. doi: 10.3389/fimmu.2026.1913000 (PMC13619957; doi:10.3389/fimmu.2026.1913000)
Supplement: Supplementary file 1 [file Table1.docx]

**Supplementary material**

**Supplementary methods**

**1. Extended DAT Testing Methodology**

The extended DAT was performed by gel column agglutination using DC-Screening I ID-Cards (DiaMed GmbH, Cressier, Switzerland, product identification 50830). The card contains five monospecific antihuman globulin reagents suspended in gel, including rabbit anti-IgG, anti-IgA, anti-IgM, and anti-C3c, as well as monoclonal anti-C3d derived from cell line C139-9, together with a negative control microcolumn. A 0.8% suspension of the patient’s unwashed red blood cells was prepared in ID-Diluent 2, a modified low ionic strength solution, by adding 10 μL of packed red blood cells to 1.0 mL of diluent. Fifty microliters of the red blood cell suspension was added to each microcolumn, followed by centrifugation for 10 minutes in an ID-Centrifuge. The reactions were visually interpreted and recorded according to the manufacturer’s instructions. The assay was considered valid only when the negative control microcolumn was nonreactive.

**2. Cold Agglutinin Titration and Thermal Amplitude Testing**

Cold agglutinin titration was performed using a conventional saline tube method. Blood samples were maintained at approximately 37°C and were not exposed to cold conditions before serum separation. Patient serum was serially diluted twofold in normal saline, and each dilution was tested with an approximately 2% suspension of saline-washed group O reagent red cells. After overnight incubation at 4°C, the cold agglutinin titer was defined as the reciprocal of the highest serum dilution showing macroscopic agglutination. A saline red-cell negative control was tested in parallel. Thermal amplitude was assessed by direct saline agglutination using the same group O reagent red cells. Reactions were incubated for 1 hour at 4°C, 22°C, 30°C, and 37°C. The highest temperature at which at least 1+ agglutination remained detectable was recorded as the thermal amplitude.

**3. Red-Cell Elution Testing**

Red-cell elution was performed using a glycine acid elution kit (Sample Release Agent, Guangzhou Zhanquan Biotechnology Co., Ltd., Guangzhou, China). DAT-positive red cells were washed four times with normal saline, and the final wash supernatant was retained as a control. Subsequently, 0.5 mL of packed red cells was mixed with 0.5 mL of acidic elution solution and centrifuged at 3000 rpm for 1 min. The recovered supernatant was neutralized with tris buffer and centrifuged again at 3000 rpm for 1 min. For antibody testing, 50 μL of the eluate and 50 μL of a 0.8% to 1% suspension of reagent red cells with known phenotypes were added to each microcolumn, incubated at 37°C for 15 min, centrifuged in a dedicated gel-card centrifuge, and interpreted according to the manufacturer’s instructions. The final wash control was tested in parallel.

**4. Transfusion compatibility testing and RBC selection**

The patient was blood group AB and RhD positive. The patient had not received an RBC transfusion within the preceding 3 months. Extended antigen phenotyping by saline technique showed reactivity as follows: anti C (3+), anti c (negative), anti E (negative), anti e (3+), anti Jka (negative), and anti Jkb (4+), consistent with a presumptive phenotype of CCee and Jk (a-b+).

Initial antibody screening using a three-cell screening panel (Guangdong Ying’an, China) and antibody identification using a 16-cell panel (Makropanel 16, Sanquin, the Netherlands) demonstrated uniform panreactivity, with a positive autocontrol (3+). The eluate prepared from the patient’s erythrocytes showed broad reactivity with reagent red blood cells. No specific alloantibody pattern could be identified in the initial testing.

Because the patient had not received recent RBC transfusion, warm autoadsorption using the patient’s erythrocytes was performed to remove autoantibody interference. Repeat antibody screening of the autoadsorbed plasma was negative, and no clinically significant alloantibody was detected.

Comparative major crossmatch testing was performed using multiple ABO-identical and RhD-compatible donor units, including donors with matched or mismatched Rh and Kidd phenotypes. All tested units demonstrated similar serologic reactivity attributable to the autoantibody, without an antigen-specific pattern suggestive of an underlying alloantibody. Therefore, RBC selection was primarily based on the exclusion of clinically significant alloantibodies and predicted extended phenotype matching, rather than on a least-incompatible approach.

ABO-identical, RhD-compatible, leukocyte-reduced RBC units matched for the patient’s documented Rh and Kidd phenotypes were selected. A total of 8 RBC units were transfused slowly through an in-line blood warmer. The patient was maintained in a warm environment and closely monitored, with no transfusion-related adverse reactions observed.

**5. Therapeutic plasma exchange**

Four therapeutic plasma exchange procedures were performed on hospital days 5, 6, 13, and 16. The target exchange volume was individualized according to the estimated plasma volume, calculated using the following formula [1]:

*Estimated plasma volume (L)=0.065×body weight (kg)×(1−hematocrit)*

The body weight and hematocrit closest to each procedure were used for calculation. Approximately one estimated plasma volume was removed during each procedure. The removed plasma volume was replaced on a 1:1 volume basis with 5% human albumin solution to maintain isovolemia. Acid-citrate-dextrose solution A was used for anticoagulation. On hospital days 5 and 6, therapeutic plasma exchange was completed before IVIG administration. No procedure-related complications were observed.

**6. Detailed treatment timeline and disease monitoring**

Intravenous ganciclovir was initiated on hospital day 3 at 5 mg/kg every 12 hours. Dexamethasone was initiated on hospital day 5 at 0.15 mg/kg/day and reduced to 0.075 mg/kg/day on hospital day 21. Intravenous immunoglobulin was administered at 0.4 g/kg/day for 5 consecutive days from hospital days 5 to 9. Therapeutic plasma exchange was performed on hospital days 5, 6, 13, and 16. On hospital days 5 and 6, plasma exchange was completed before IVIG infusion. No procedure-related complications were observed. The patient received 4 units of leukocyte-reduced red blood cells on each of hospital days 5 and 6, for a total of 8 units.

Serial hemoglobin, bilirubin, lactate dehydrogenase measurements Haptoglobin and absolute reticulocyte count are presented in the revised Figure 2 and Table S2. Extended DAT at diagnosis demonstrated IgG 4+, IgA 4+, IgM 3+, and C3d 3+ reactivity. On hospital day 21, only IgG remained positive at 4+, whereas IgA, IgM, C3c, and C3d were negative.

**References**

1. Kaplan AA. A simple and accurate method for prescribing plasma exchange. ASAIO Trans. (1990) 36:M597-M599.

| **Table S1.** Additional Workup of Hemolytic Anemia. | |
| --- | --- |
| **Investigation** | **Result** |
| CT Thorax/Abdomen/Pelvis | No evidence of lymphoma |
| Bone marrow morphology | Bone marrow smear showed active hematopoiesis with marked erythroid hyperplasia, relative granulocytic reduction, reversal of the myeloid-to-erythroid (M:E) ratio |
| Bone marrow flow cytometry | No immunophenotypically abnormal cell population was detected |
| Bone marrow biopsy | Bone marrow biopsy revealed normal cellularity (approximately 40%) with a decreased myeloid-to-erythroid (M:E) ratio. Granulocytic and erythroid precursors were predominantly at intermediate stages of maturation, and megakaryocytes showed predominantly lobulated nuclei. No fibrosis or malignant infiltration was identified |
| Other investigations | The activity of glucose-6-phosphate dehydrogenase was normal; No PNH clone was detected |

Abbreviations: CT, computed tomography; PNH, paroxysmal nocturnal hemoglobinuria.

**Table S2.** Treatment timeline and serial hemolysis-related laboratory findings during hospitalization.

|  |  |  | **Intravenous ganciclovir** 5 mg/kg every 12 hours on Days 3-25 | | | | | | | | |
| --- | --- | --- | --- | --- | --- | --- | --- | --- | --- | --- | --- |
|  |  |  |  |  | **Dexamethasone** 0.15 mg/kg/day on Days 5-20, then 0.075 mg/kg/day on Days 21-25 | | | | | | |
|  |  |  |  |  | Plasma exchange | Plasma exchange |  | Plasma exchange | Plasma exchange |  |  |
|  |  |  |  |  | Transfused 4U RBCs | Transfused 4U RBCs |  |  |  |  |  |
|  |  |  |  |  | IVIG 0.4 g/kg/day on Days 5-9 (post-Plasma exchange) | | |  |  |  |  |
| **Parameters** | **Reference range** | **Day 1** | **Day 3** | **Day 4** | **Day 5** | **Day 6** | **Day 9** | **Day 13** | **Day 16** | **Day 21** | **Day 25** |
| Hb, g/L | 130-170 | 127 | - | 40 | 32 | 69 | 104 | 74 | 90 | 107 | 113 |
| LDH, U/L | <248 | 2982 | - | 3535 | 8466 | 3349 | 1895 | 1609 | 809 | 659 | 297 |
| ARC, ×10⁹/L | 24-84 | - | - | 145 | 182.1 | 207 | 162.4 | 173.7 | 149.2 | 126.9 | 132.6 |
| TBIL, μmol/L | 1.7-20.0 | 485.8 | 318.2 | 926 | - | 760 | 536 | 338.3 | 230 | 155.5 | 100.3 |
| IBIL, μmol/L | 1.7-16.0 | 201.7 | 160.3 | 498.6 | - | 322.4 | 219.4 | 128.8 | 95.9 | 26.8 | 16 |
| HPT, g/L | 0.2-2.0 | - |  | <0.2 | <0.2 | - | - | <0.2 | - | <0.2 | 0.4 |
| Extended DAT | - | - |  | IgG (4+), IgA (4+), IgM (3+), C3d (3+) | - | - | - | - | - | IgG (4+) | - |

Abbreviations: Hb, hemoglobin; LDH, lactate dehydrogenase; ARC, absolute reticulocyte count; TBIL, total bilirubin; IBIL, indirect bilirubin; HPT, haptoglobin; DAT, direct antiglobulin test; IVIG, intravenous immunoglobulin; RBCs, red blood cells.
